# Leaving School: A Healthy Transition in Late Adolescence?

**DOI:** 10.3390/ejihpe15080146

**Published:** 2025-07-25

**Authors:** Max Herke

**Affiliations:** Institute of Medical Sociology, Medical Faculty, Martin Luther University Halle-Wittenberg, 06112 Halle (Saale), Germany; max.herke@posteo.de

**Keywords:** subjective well-being, adolescence, leaving school, Germany, National Educational Panel Study

## Abstract

Background: Adolescents’ subjective well-being (SWB) is a key indicator of quality of life. While its development during schooling has been widely studied, few studies have examined changes in SWB after leaving school due to the need for longitudinal data. This study investigates changes in SWB among adolescents in Germany over the two years before and after leaving school, focusing on school type, socioeconomic position, gender, and family structure. Methods: We use data from the ninth-grade cohort of the German National Educational Panel Study, first surveyed in 2010 and followed annually. Growth modeling (specifically, a multilevel discontinuity model) is applied to analyze SWB trajectories and potential moderation by background characteristics. The final sample includes 19,767 observations from 6599 individuals. Results: SWB increases notably after leaving school and remains stable before and after the transition. The increase is smaller for adolescents completing higher secondary education, living in nuclear families, or identifying as male. These groups report higher SWB prior to the transition, so post-school changes reduce group differences. Conclusion: The findings suggest that schools may lack adequate resources to support adolescents in mastering key developmental challenges. While school is a critical environment, it may also impose pressures that are associated with lower well-being.

## 1. Introduction

In the last three decades, the well-being of adolescents in Europe has been extensively researched in large-scale, cross-national studies such as the Health Behaviour in School-aged Children (HBSC) surveys (Cantril, 1965; Cavallo et al., 2015; Elgar et al., 2015; Inchley et al., 2016) and many smaller projects. Subjective well-being is an important indicator of quality of life and closely linked to current and future subjective health (Hoyt et al., 2012; Sawyer et al., 2012). It is the result of an overall experience of the individual, measured by a multidimensional assessment that reflects the combination of cognitive and affective processes for children, adolescents, and adults (Diener et al., 1999). The cognitive processes cover various specific areas (e.g., education, health, family, and friends). The affective processes include both the presence of positive emotional states (e.g., happiness and self-esteem) and the absence of negative emotions (depression, anxiety) (Diener et al., 1999; Hoyt et al., 2012). The findings attest to good or even excellent well-being for children and adolescents in schools but must be interpreted in the light of further findings. First, well-being significantly decreases during the course of the school career (Proctor et al., 2009; Herke et al., 2019). Secondly, significant health inequalities are already evident at this phase in life (Cavallo et al., 2015; Elgar et al., 2015). And thirdly, although adolescent well-being along the school career is well studied, the impact of leaving school is not (Lefkowitz, 2005; Salmela-Aro & Tuominen-Soini, 2010; Siembab & Stawarz, 2019).

Adolescence is accompanied by numerous developmental tasks; physical, emotional, cognitive, and social changes and transitions bring with them demanding challenges as well as risks (Patton & Viner, 2007; Proctor et al., 2009; Eccles & Roeser, 2011; Sawyer et al., 2012). The successful accomplishment of these developmental challenges or developmental tasks (Hurrelmann & Bauer, 2015) is conducive to both present and future health, and, conversely, good health can contribute to completing these tasks (Hoyt et al., 2012; Sawyer et al., 2012; Hurrelmann, 2016). These developments are also taking place in the context of school, which plays a central role in the lives of young people and for the accomplishment of developmental tasks. Thus, young people have to cope with the demands of school and the development of professional perspectives linked to it, and at the same time, there is an increasing separation from the parental home, the development of new social networks, individualization processes, and the development of social relationships with peers (Eccles & Roeser, 2011; Hurrelmann, 2012).

However, previous findings indicate both an increase in pressure to perform and a deterioration in motivation, school attachment, academic achievement, and well-being at school, especially throughout secondary education (Eccles & Roeser, 2011; Hascher & Hagenauer, 2011). This particularly affects students in upper secondary education, who are being prepared for tertiary education and the demands that go with it (Lefkowitz, 2005). The “Stage-Environment-Fit” theory states that discrepancies between requirements to accomplish developmental tasks and resources provided by the environment are associated with negative consequences (Eccles & Roeser, 2009). The theory compares whether the needs of the adolescent’s current stage of development (“stage”) with what is provided by the school context (“environment”) fit together (“fit”). In the context of secondary education, several aspects of the school environment may increasingly misalign with adolescents’ developmental needs. These include rising academic pressure, limited autonomy, and intensified social comparison among peers. Academic curricula become more performance-oriented, often focusing on high-stakes exams and long-term consequences for higher education access. Simultaneously, opportunities for self-determined learning and individual decision-making remain scarce. Peer environments may become more competitive, and adolescents become more aware of social hierarchies and achievement differences. According to stage-environment-fit theory, such discrepancies are likely to impair motivation, self-esteem, and ultimately subjective well-being. Leaving school may relieve adolescents from these misalignments and restore a sense of autonomy and personal control, which are core components of psychological well-being. However, this relief may come at the cost of reduced educational opportunities and long-term disadvantages, particularly for students exiting lower secondary education without qualifications for tertiary education. Prior research has shown that lower educational attainment is associated with poorer health, reduced income, and increased social risks in adulthood (Viner et al., 2012).

In upper secondary education in particular, the demands increase sharply, and adolescents will become more aware of interindividual differences in skills and performance (Salmela-Aro et al., 2008, 2009). This burnout at school can be defined as exhaustion due to school demands, negative and distanced attitudes towards one’s own school, and feelings of inadequacy as a student (Salmela-Aro et al., 2008). With regard to the stage-environment-fit theory, it can also be stated that coping with the demands of school performance not only fails to meet the needs of the adolescent’s developmental stage but is also so heavy a burden in its own right that leaving school could lead to improved well-being. According to the stage-environment-fit theory (Dijkstra et al., 2008; Eccles & Roeser, 2009; Booth & Gerard, 2014), the type of educational environment is more important for changing the life satisfaction of young people than the transition itself (Salmela-Aro & Tuominen-Soini, 2010).

Based on this framework, we expect that subjective well-being will increase after leaving school, particularly for adolescents who experienced a poor fit between their developmental needs and the school environment. As adolescents progress through secondary education, they often face increasing academic pressure, limited autonomy, and intense peer comparison. These demands can lead to emotional exhaustion, disengagement, and diminished self-worth. Leaving school may relieve adolescents from these misalignments and restore a sense of autonomy and personal control, which are core components of psychological well-being. Therefore, we hypothesize that subjective well-being improves once adolescents leave the school context, especially for those most affected by school-related stressors.

Many other resources are important for the accomplishment of developmental tasks, and the availability of resources is strongly linked to socio-economic status. Traditionally, the socioeconomic status of children and adolescents is determined by various indicators related to their origin, such as parental education, household income, family wealth, or a combination of different characteristics (Richter, 2005; Hagquist, 2007). As they enter adolescence, adolescents also begin to develop a “separate” social status (Koivusilta et al., 2006; Hagquist, 2007; Havas et al., 2009). In this context, national and international studies show that the educational status of adolescents—often measured by their performance at school or the type of school attended—plays an equally important, or in some cases even more important, role for health and health behavior than indicators of the social status of the family of origin (Havas et al., 2009; Lampert, 2010; Kuntz & Lampert, 2013; Moor & Richter, 2013). However, the family is also relevant beyond socio-economic status as a resource for developmental tasks. It is crucial for the health of its children throughout their lives (Ben-Shlomo & Kuh, 2002; Hayward & Gorman, 2004; Galobardes et al., 2008). Children and adolescents from socially disadvantaged families generally grow up under less favorable living conditions and have correspondingly worse health opportunities than their peers from socially better-off families (Starfield et al., 2002; Inchley et al., 2016). Thus, when investigating the influence of the school context on adolescent health, other individual factors must be considered and controlled, as they influence health and may be associated with school characteristics.

The aim of this study is to analyze the changes in well-being of adolescents when leaving school, how the impact of this transition might be moderated by social determinants of health, and if health inequalities change as well. Using panel data from the National Educational Panel Study (NEPS) (Blossfeld et al., 2011), we analyze the trajectories of well-being over four time points: two before and two after leaving school, each one year apart. This design is chosen to assess whether there is a relatively stable change. Apart from the transition, we account for a change in well-being over time in general and examine the impact of several indicators of socioeconomic position, as well as gender and family structure, independently and as moderators of the impact of the transition. We do not, however, focus on the context adolescents enter after leaving school. The following questions are the focus of this study:

1. How does adolescent well-being change when leaving school?
2. How is this change moderated by socioeconomic position and other background characteristics?

We assume that subjective well-being increases after leaving school. Furthermore, we assume the increase to be higher for students leaving higher secondary education, due to the greater performance pressure, and smaller for those in advantaged socioeconomic positions or living in nuclear families, due to the available material or social resources possibly mitigating the impact of school stressors.

## 2. Materials and Methods

### 2.1. Sample

This work uses data from the German National Educational Panel Study (NEPS), which collects longitudinal data on the development of competencies, educational processes, educational decisions, and returns to education throughout the life span (Blossfeld et al., 2011). NEPS is comprised of six distinct cohorts sampled from different age groups. This study focuses on data from starting cohort four (SC4), which started with a representative sample of German 9th graders in 2010 and was followed up annually or biannually (Aßmann et al., 2011). Depending on their educational track, participants left school after grades 9 to 13, typically between the ages of 15 and 19. Those in the vocational track exited earlier, while those in the academic track graduated later. While our models do not directly use grade level, we account for variation in school-leaving age through a design that aligns each respondent’s data to their individual transition point.

NEPS employed a multistage sampling approach. Schools were first sampled based on strata such as school type and region, followed by a selection of classes and inclusion of all students within those classes. In cases of school non-participation, structurally similar replacements were drawn (Aßmann et al., 2011). Surveys were initially conducted via paper questionnaires administered in classroom settings. Additional information was collected through telephone interviews with parents and paper-based questionnaires for teachers and school administrators. If students remained in their original schools, follow-ups were paper-based; otherwise, interviews were conducted by phone. Parents, teachers, and principals were re-interviewed at intervals of one to three years. All NEPS data include unique identifiers for individuals, classes, and institutions across waves, allowing longitudinal data linkage. In this study, student-reported information is supplemented with parent-reported indicators, which are treated as individual-level variables.

An overview of available observations with complete data in all included variables at each time of measurement is given in Table 1.

### 2.2. Indicators

Subjective well-being was assessed using a modified version of the Personal Well-being Index—School Children (Cummins & Lau, 2005; Tomyn & Cummins, 2011). Respondents rated their satisfaction across six domains: life satisfaction (“How satisfied are you with your life overall at the present?”), standard of living (“…with what you have? Think of money and things that you own.”), personal health (“…with your health?”), family (“…with your family life?”), personal relationships (“…with your group of friends and acquaintances?”) and school (“…with your situation at school?”). Each item was rated on a scale from 0 (“not satisfied at all”) to 10 (“completely satisfied”) (Cantril, 1965). Scores were combined into a summary index and linearly rescaled to range from 0 to 100.

Transition and time are the main explanatory variables. Transition is a dichotomous variable indicating whether a student still attends school (0) or has left school (1). Time indicates the measurement point (1–4), which represents the last two school years and the two years after leaving school at annual intervals. This is complemented by school type, parental education, and household income to examine health inequalities, as well as gender and family structure as controls. Importantly, the transition variable is defined at the individual level and does not correspond to a fixed measurement wave. Adolescents differ in the timing of school leaving depending on their educational track, and each participant contributes both pre- and post-transition observations. Each participant contributes four observations: two before and two after leaving school. “Time” refers to the number of years before or after the transition (coded as 1 to 4), irrespective of the specific survey wave or chronological age. This design allows for a within-person estimation of the transition effect, reducing the risk that the observed increase in well-being merely reflects general age-related maturation rather than the act of leaving school itself.

School type is included as a dichotomous measure, distinguishing either students following an academic track, i.e., those attending higher secondary education necessary for entry into university and tertiary education, or students on a vocational track, i.e., those leaving school after lower secondary education. The most commonly distinguished school types in the hierarchically organized German school system are the following: The “Gymnasium” provides the most frequent academic track and ends after grade 12 or 13. The “Realschule” ends after grade 10, and the “Hauptschule” ends after grade 9 or 10, respectively. Vocational tracks end after lower secondary education. The “Gesamtschule” offers both tracks, and students can follow either an academic or a vocational track. School type is kept constant for all time points; students changing school types were excluded.

Parental education and household income are derived from data from the parent interviews. Parental education is the highest educational level attained by a parent on the CASMIN-classification (Comparative Analysis of Social Mobility in Industrial Nations) (Brauns et al., 2003). We differentiate high (at least university entrance qualification or higher) and low (all other educational attainments) parental education. Household income uses the squared scale for equivalized household income (OECD, 2008) and differentiates high and low household income, split by the median.

Information on gender and family composition was obtained from the adolescent respondents themselves. Participants were asked to indicate their gender and to report which individuals lived in their primary household, including mother, father, stepmother, stepfather, siblings, or others. Based on this information, a binary classification of family structure was constructed. Adolescents residing with both biological parents were categorized as living in a nuclear family, while all other constellations—including single-parent households, stepfamilies, or other arrangements—were grouped as alternative family structures.

Parental education, household income, and family structure were not assessed in every wave. When data were missing at a given wave, we carried forward the most recently available information for the respective individual. This approach was chosen to maximize sample size while maintaining comparability across time points. However, in cases where these characteristics changed during the study period, such changes were not captured in our models. A description of the sample and distribution of characteristics at the first time point is given in Table 1.

### 2.3. Statistical Analysis

First, we present trends of subjective well-being grouped by time of measurement and also by school type, parental education, household income, gender, and family structure. We show the mean values of subjective well-being, complemented by the 95% confidence intervals around the means.

Second, we conduct multivariate analyses using multilevel discontinuity models, which allow for estimating within-person changes in subjective well-being associated with school leaving. Rather than modeling individual growth trajectories with latent slope factors, our approach captures average within-person changes by including a binary indicator for school leaving (transition) and a linear time trend. This structure is particularly suited to evaluate discontinuous changes in subjective well-being that occur at different times for different individuals (Bollen & Curran, 2006; de Leeuw & Meijer, 2008; Curran et al., 2010). The growth model includes the dichotomous indicator for the transition as well as time of measurement and examines general changes independent of leaving school. School type, parental education, household income, gender, and family structure are included as independent variables, and three models are computed. Model 1 (M1) includes all variables. Model 2 (M2) adds interactions between school type, parental education, household income, gender, and family structure with the indicator for the transition. Model 3 (M3) is a reduced model that only keeps terms that are significant in M1 or M2. The analyses will be complemented by sensitivity analyses, which modify indicators and models to test the robustness of the results.

The statistical analyses were conducted with the free statistical software R version 4.3.2, and the R packages “ggplot2” (Wickman, 2016) and “lme4” (Bates et al., 2015) were used for the preparation of figures and multilevel modeling, respectively. The syntax used for the statistical analyses is available from the author upon request.

## 3. Results

### 3.1. Descriptive Results

**Figure 1:**
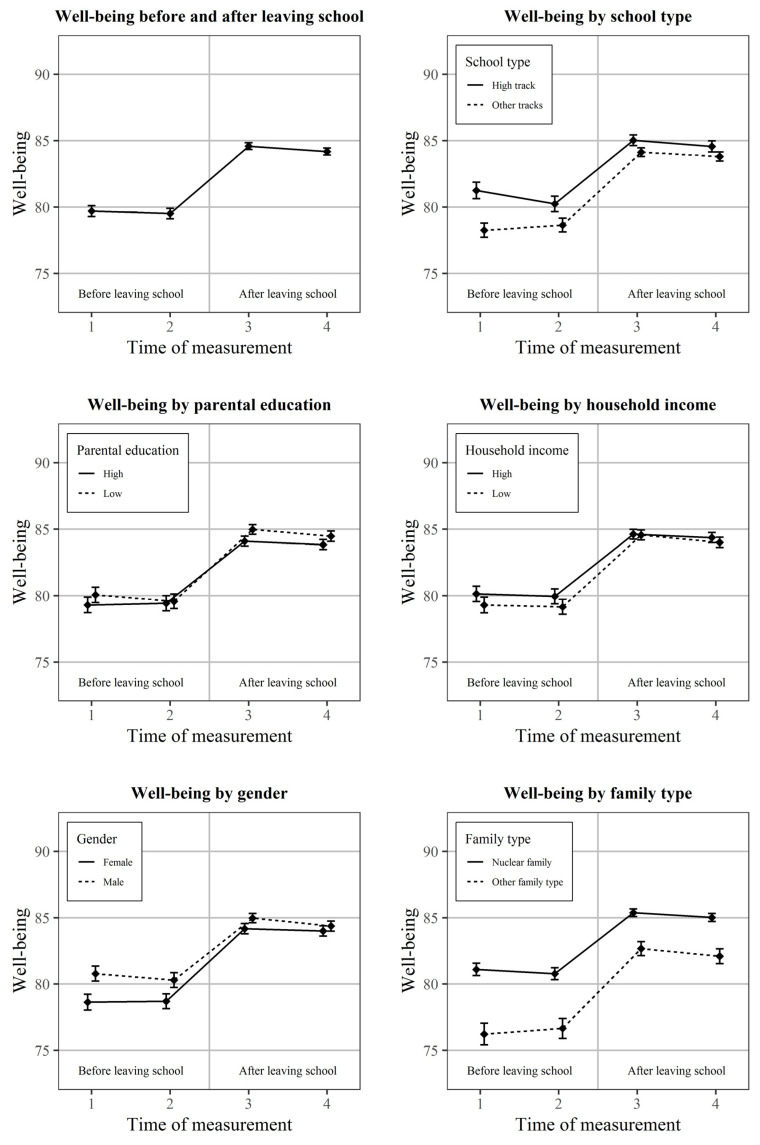
Development of subjective well-being of adolescents two years before and two years after leaving school in Germany (starting cohort four of the National Educational Panel Study). Mean values of subjective well-being of 19,767 observations of 6599 individuals over four time points, complemented by 95% confidence intervals.

The changes in subjective well-being of adolescents in the two years before and the two years after leaving school are presented in Figure 1. The dependent variable subjective well-being is scaled to range from 0 to 100, with higher values indicating better subjective well-being. There is a noticeable improvement in subjective well-being by 5.2 points between time points 2 and 3, when adolescents leave school. A paired t-test reports a significant difference in this change (t(3815) = 24.93, *p* < 0.001).

A comparison the average subjective well-being over all time points grouped by the other independent variables shows only small differences. The reported subjective well-being is generally higher for those adolescents who attended the academic track (1.5 points), who have parents with lower educational attainment (0.6 points), who have high household income (0.6 points), who live in a nuclear family (3.8 points), or who are male (1.3 points). Whether these characteristics have a significant impact on subjective well-being and if they moderate the impact of the transition are tested in the following regression models.

### 3.2. Multivariate Results

Results of the growth models are presented in Table 2. Again, the dependent variable subjective well-being is scaled to have a range from 0 to 100, with higher values indicating better subjective well-being. The coefficient for transition indicates the change in subjective well-being when leaving school, and the coefficient for time indicates all other linear changes in the dependent variable. All other fixed effects indicate average differences in subjective well-being over all time points. The interactions, however, indicate differential changes when leaving school.

M1 shows that adolescents’ subjective well-being increases significantly and is 5.7 points higher in the two years after leaving school than in the two years prior to it. Additionally, there is a slight decrease in subjective well-being over time, underlying the impact of the transition. Furthermore, the multilevel regression models support the differences found in the bivariate analyses. This means that those adolescents who attended the academic track, have parents with lower educational attainment, have high household income, live in a nuclear family, or are male report significantly better subjective well-being.

M2 shows significant interactions for school type, gender, and family structure. In this model, the increase in subjective well-being after leaving school rises to 6.2 points. The interactions show that adolescents who attended the vocational track, who do not live in nuclear families, or who are female have a greater increase in subjective well-being when leaving school. All other indicators show an impact similar to M1, with changes in magnitude of effects but not direction.

Finally, M3 retains only significant effects and shows a similar picture to M2. The increase in subjective well-being from leaving school is even greater at 6.9 points. Log-likelihood-based tests show that M2 and M3 are each superior to M1, but M3 is not significantly better than M2.

### 3.3. Sensitivity Analyses

Several sensitivity analyses mostly replicate these results. A split by school type, i.e., analyzing adolescents from the academic and the vocational tracks independently, does not reproduce the direct impact of gender for those from the academic track and of parental education for both tracks. Splits by gender or family type reproduce all the findings for male adolescents or those living in nuclear families, but not the direct impact of parental education or household income for female adolescents or those not living in nuclear families. All interactions, however, are reproduced in these split analyses, if applicable. Furthermore, using only time points 2 and 3 does not reproduce the differential impact of leaving school by school type. Finally, an additional variable indicating the context into which adolescents are transitioning, i.e., university, vocational training, or work, does not provide consistent differences by the context entered, which might be due to a lack of granularity in the available indicator.

## 4. Discussion

This study examines the changes in subjective well-being of adolescents leaving school and shows a considerable increase following the transition. Levels of subjective well-being are relatively stable in the two years prior and the two years after leaving school. This change is moderated by several factors. There is a smaller increase for those adolescents who leave school after the academic track, who live in nuclear families, and who are male. These adolescents, however, have greater subjective well-being before the transition, so leaving school decreases the differences.

The increase in subjective well-being after leaving school is generally in line with the few existing studies (Lefkowitz, 2005; Salmela-Aro et al., 2008, 2009; Salmela-Aro & Tuominen-Soini, 2010; Siembab & Stawarz, 2019) and is consistent with the stage-environment-fit theory (Eccles & Roeser, 2009). While developmental maturation may partly explain improved well-being in late adolescence, our within-person transition model strongly suggests that the act of leaving school itself plays a central role in the observed change.

Considering findings on the decrease in subjective well-being throughout secondary education, which is even more pronounced in those adolescents attending academic track school types (Herke et al., 2019), as well as the sharp increase in subjective well-being when leaving school found in this study, supports the assumption that the school context may lack adequate resources to support adolescents in mastering developmental tasks. The fact that those attending vocational tracks show greater improvement is contrary to our initial assumption.

The assumed higher performance pressure and demands in higher secondary education might be overcompensated for by the substantially better subjective well-being of students entering the academic track in earlier grades (Herke et al., 2019) and may also be the results of health inequalities, as school type is a strong indicator for the adolescents’ own socioeconomic position in Germany, and those attending academic tracks are usually favored in terms of personal and material resources. Similarly, the greater increase in subjective well-being for adolescents living in non-nuclear families might be due to lower levels of social support, which may impair their capacity to master developmental tasks within the school context. A comparable dynamic may explain the stronger effect among girls, as prior studies suggest that female students often report higher levels of academic stress, emotional burden, and sensitivity to peer dynamics during adolescence (González-Carrasco et al., 2017). In this context, leaving school may represent a more profound relief for girls, reducing multiple school-related stressors at once. This gendered effect highlights the need for further research into how subjective well-being is shaped by both structural and emotional dimensions of the school environment.

In the socialization model, the focus is on coping with developmental tasks, since successful or unsuccessful coping is considered crucial for the development of health and illness. In order to successfully cope with developmental tasks, productive reality processing must succeed. This consists of constantly moderating all aspects of the developmental tasks—physical, psychological, social, and ecological requirements—and coordinating the resulting impulses with each other in order to initiate a positive development of health. If this does not succeed, this in turn contributes to the dynamics of illness. The existing personal and social conditions and resources are also relevant for the accomplishment of the developmental tasks (Hurrelmann, 2012; Hurrelmann & Bauer, 2015). The school provides a context that contributes as a resource to mastering developmental tasks and within which comparison processes with peers take place, which influence school satisfaction and thus have a direct effect on health and well-being through the unsuccessful mastering of developmental tasks or, indirectly, through a negative influence on self-concept, self-esteem, and school well-being. However, research on positive emotions and well-being at school also shows that, in addition to contextual factors, numerous individual factors are of course also relevant, including other school-related characteristics such as school performance (Hascher & Edlinger, 2009).

In the course of secondary education, the school demands increase steadily, and adolescents will become more aware of inter-individual differences in skills and performance (Salmela-Aro et al., 2008, 2009). In accordance with the stage-environment-fit theory (Eccles & Roeser, 2009), in this case, the adaptation of the school context to the needs of adolescents will continue to decline, which in turn hinders successful coping with developmental tasks, leading to increasing stress and a deterioration in health dynamics. Schools of the vocational track without upper secondary level, which primarily prepare students for vocational training after leaving school, on the other hand, offer lower demands and a safer, more supportive environment (Deci & Ryan, 2002). This is in line with findings on the decrease of subjective well-being throughout secondary education but ultimately does not fully compensate for the health inequalities favoring students in the academic track.

Key strengths of this study are based on the longitudinal design made possible by the high-quality panel data of the NEPS, which allows examining intra-individual changes, as opposed to mere trends, and the exceptional robustness of findings. However, some limitations of this study should be noted as well. Extensive sensitivity showed the robustness and reproducibility of most results. Furthermore, while most independent variables are unlikely to change, and adolescents changing school types were excluded, changes in socioeconomic position or family structure are not rare. A further limitation is the treatment of these background characteristics as time-invariant when data were missing. Although this approach ensures a complete dataset, it may overlook meaningful changes in adolescents’ living conditions that could influence their well-being. Experiencing such transitions can have a distinct influence on adolescents’ subjective well-being, but these were not explicitly considered in the model or interpretation, as these should be examined in dedicated analyses that take into account further aspects, e.g., timing and transitional periods. Still, including time-varying covariates in the model should not introduce significant bias (Salmela-Aro & Tuominen-Soini, 2010; Cavallo et al., 2015). Although we did not conduct a specific analysis of differential dropout in this study, prior research and extensive experience with the NEPS dataset suggest that attrition is primarily associated with school track and socioeconomic characteristics. As our models account for these variables, the risk of substantial attrition bias is likely low.

Importantly, the present study should not be interpreted as advocating for early school leaving. While subjective well-being improves after adolescents leave school, this likely reflects a release from situational stressors within the school environment rather than the intrinsic benefits of leaving education. As prior research has consistently shown, educational attainment is one of the strongest predictors of health, well-being, and life chances in the long run. Our findings should therefore be seen as a call for educational systems to improve their developmental fit with adolescents’ needs, rather than as support for early exit as a coping strategy.

Our findings suggest that schools may not adequately support adolescents in managing the multiple developmental challenges they face. While schools are key social environments during adolescence, they may also place growing demands on students and contribute to stress and reduced well-being. Improving the school context to better meet adolescents’ needs could play a vital role in promoting long-term health and development.

## Figures and Tables

**Table 1:** Sample description (data from the starting cohort four “Ninth graders” of the National Educational Panel Study).

**Observations**	**Time point**	**Number of complete cases**
	t1	5200
	t2	5274
	t3	4718
	t4	4575
**Dependent variable**	**Time point**	**Mean and standard error**
Subjective well-being	t1	79.7 (0.21)
	t2	79.5 (0.20)
	t3	84.6 (0.13)
	t4	84.2 (0.14)
**Independent variables**	**Value**	**Relative frequency**
School track	Academic track	51.7%
	Vocational track	48.3%
Gender	Female	50.3%
	Male	49.7%
Parental Education	High	46.8%
	Low	53.2%
Parental income	High	48.6%
	Low	51.4%
Family structure	Nuclear family	71.4%
	Other family types	28.6%

**Table 2:** Growth models (19,767 observations of 6599 individuals over four time points).

	M1		M2		M3	
**Fixed Effects**	**Beta**	**SE**	**Beta**	**SE**	**Beta**	**SE**
Intercept	77.90 ***	0.40	77.48 ***	0.53	76.98 ***	0.48
School track Ref = academic track	−1.42 ***	0.23	−2.38 ***	0.33	−2.17 ***	0.32
Gender Ref = female	0.69 **	0.22	1.67 ***	0.32	1.68 ***	0.32
Parental education Ref = high	0.70 **	0.25	0.32	0.35	0.64 *	0.25
Parental income Ref = high	−0.53 *	0.23	−0.89 **	0.32	−0.56 *	0.23
Family structure Ref = nuclear family	−3.35 ***	0.23	−4.49 ***	0.34	−4.56 ***	0.34
Time	−0.37 **	0.13	−0.36 **	0.13	−0.37 **	0.13
Transition	5.65 ***	0.29	6.20 ***	0.51	6.91 ***	0.41
Transition × school track Ref = academic track	-		1.34 ***	0.32	1.04 ***	0.29
Transition × gender Ref = female	-		−1.28 ***	0.29	−1.30 ***	0.29
Transition × parental income Ref = high	-		0.43	0.34	-	
Transition × parental education Ref = high	-		0.46	0.32	-	
Transition × family structure Ref = nuclear family	-		1.52 ***	0.33	1.61 ***	0.32
**Random Effects**	**Variance**		**Variance**		**Variance**	
Intercept	128.77		220.17		220.30	
Transition	43.35		8.12		8.12	
Residual	73.61		74.30		74.32	
Log-Likelihood	−75,307.64	(df = 12)	−75,424.01	(df = 17)	−75,426.17	(df = 15)

Notes: Ref = reference category; SE = standard error; df = degrees of freedom; Significance levels: * = *p* < 0.05, ** = *p* < 0.01, *** = *p* < 0.001.

## Data Availability

The data that support the findings of this study are not publicly available due to data protection regulations and contractual restrictions with the data provider. The authors do not have permission to share the data. Access to this data requires application and approval by the data provider.

## Abbreviations

The following abbreviations are used in this manuscript:

SWB	subjective well-being
NEPS	National Educational Panel Study
